# Biological and Physicochemical Assessment of Middle Ear Prosthesis

**DOI:** 10.3390/polym11010079

**Published:** 2019-01-06

**Authors:** Magdalena Ziąbka, Michał Dziadek, Aleksandra Królicka

**Affiliations:** 1 AGH University of Science and Technology, Faculty of Materials Science and Ceramics, Department of Ceramics and Refractories, 30-059 Krakow, Poland; 2 AGH University of Science and Technology, Faculty of Materials Science and Ceramics, Department of Glass Technology and Amorphous Coatings, 30-059 Krakow, Poland; dziadek@agh.edu.pl; 3 University of Gdansk, Intercollegiate Faculty of Biotechnology UG-GUMed, Department of Biotechnology, Laboratory of Biologically Active Compounds, 80-307 Gdansk, Poland; aleksandra.krolicka@biotech.ug.edu.pl

**Keywords:** polymeric implants, middle ear prosthesis, bactericidal efficacy, cytotoxicity

## Abstract

Polymers modified with bioactive nanoparticles are a promising solution for patients who need a tissue replacement. Modern implants, thanks to bioactive and bactericidal functions, facilitate the healing and regeneration process of the replaced tissue. The aim of this study was to assess whether silver nanoparticles (AgNPs) could support antibacterial function without cytotoxic effect and deterioration of biostability. This article describes biological and physiochemical aspects concerning a new polymeric middle ear implant (Otoimplant) enriched with silver nanoparticles. This kind of prosthesis is a promising implant for the reconstruction of ossicles in ossiculoplasty. We found that incorporation of silver nanoparticles into a polymeric matrix resulted in bactericidal efficacy against Gram-positive and Gram-negative bacteria, both resistant to antibiotics and basic strains. Our prostheses do not show cytotoxic effect and are a suitable biomaterial platform for effective culture of Saos2 and NHOst osteoblastic cells. The in vitro incubation of the samples in distilled water revealed that surface parameters, such as roughness, may slightly increase as a result of unveiling nanoparticles. However, the prolonged immersion does not change mechanical parameters. During one-year incubation, the prosthesis proved to retain stable values of Young’s modulus, tensile strength, propagation of longitudinal ultrasonic waves, pH, and conductivity.

## 1. Introduction

Polymeric materials provide a wide range of applications as implants in different parts of the human body. Therefore, an appropriate selection of the materials that will be used for such medical devices is a key factor for a long-term success of proper implant functioning. The human body hardly ever fully accepts a foreign body—which is why any implanted material should be optimized so as to perform the assumed functions as well as to limit the adverse biologic response [1].

The ideal material for middle ear prosthesis should be biocompatible, stable, and safe. Additionally, it ought to be easily insertable and capable of yielding optimal sound transmission [2]. The technological potential of such new implant materials is also associated with the development of novel medical treatments. Some solutions may prevent infections or other diseases caused by bacteria growth and biofilm formation, while the others may promote cells adhesion, proliferation, or differentiation [3]. In the area of head and neck surgery, a risk of bacterial infection is very common [4,5]. Untreated infections lead to damage of the eardrum, the ossicles, or the hearing nerve, and—as a natural consequence—may even result in conductive hearing loss. Besides inflammatory processes accompanied by middle ear effusion or scarring, there are other illnesses leading to conductive hearing loss, such as otosclerosis, otitis media, or cholesteatoma [6,7]. Otitis media or acute otitis media are the most common conditions caused by bacteria [8]. Antibiotics are the most often used means of treating the infections related to both Gram-positive and Gram-negative bacteria. Yet for many patients suffering from acute otitis media, such a treatment is not always successful as some organisms may exhibit antibiotic resistance through beta-lactamase production [9,10].

Another approach to fight against bacteria is applying nanoparticles (NPs) of metals such as gold, copper, and silver. The antibacterial mechanisms of NPs have not been fully understood yet. Nevertheless, the phenomena that have already been described include oxidative stress induction, metal ion release, and non-oxidative mechanisms [11]. Novel properties of NPs result from their uniquely small size, which leads to a better interaction with cells due to a larger surface area-to-mass ratio and versatile and controllable application [12]. Therefore, nanoparticles are increasingly used as an antibacterial coating of implantable devices in cardiovascular apparatuses, catheters, wound dressings, bone cement, dental materials, and surgical implants [13,14,15,16,17]. Nowadays, titanium, polymer, or ceramic prostheses are most often used for reconstructing the ossicles. However, none of these prostheses show a bactericidal effect [18,19,20]. The modification of laryngological implants with silver nanoparticles aims to improve the biological properties of prostheses by endowing them with an additional bactericidal function, as described in our earlier work [21]. However, it should be remembered that bactericidal activity may simultaneously cause cytotoxic effects. Therefore, it is extremely important to ensure that implantable prostheses for middle ear reconstruction guarantee an adequate long-term biocompatibility and bio-functionality, as well as growth of a functional tissue formation, considering the specific physiological and mechanical requirements of the replaced tissue [22,23]. Still, as the lack of cytotoxicity does not necessarily provide biocompatibility [3], in this article, the authors would like to present biological and physicochemical properties of middle ear prostheses in a long-lasting experiment.

## 2. Materials and Methods

### 2.1. Material Manufacturing

The middle ear prostheses and all the investigated samples (discs for biological study and paddles for physicochemical investigations) were prepared using extrusion and injection moulding technology according to Ziąbka et al. [21]. For that purpose, commercially available acrylonitrile-butadiene-styrene copolymer (ABS) (INEOS Styrolution, Frankfurt, Germany) was prepared and dried in the laboratory dryer at 80 °C for six hours. Next, the silver nanoparticles in the amount of 0.1% wt. were incorporated and homogenized with polymer granules in the plasticizing chamber using a 0.8 m long screw. The spherical silver nanoparticles sized below 50 nm were developed at the Intercollegiate Faculty of Biotechnology, University of Gdansk and Medical University of Gdansk, manufactured according to Banasiuk et al., 2016 [24]. Subsequently, the material was injected into the steel moulding form, cooled, and extracted. The injection parameters were selected and adapted for the process according to the characteristic data sheet of polymer manufacturer (injection temperature in three zones 240 °C, injection pressure 80 kg/cm^2^, flow 70%).

### 2.2. Material Evaluation

The stability of the materials was assessed via incubation in deionized water (diH_2_O) at 37 °C for 3, 6, and 12 months. The ratio of the sample weight to diH_2_O volume was 0.1 g mL^−1^, according to PN-EN ISO 10993-13 [25]. The materials before and after incubation were analysed in terms of microstructure, surface wettability, and roughness, as well as mechanical properties. Furthermore, the pH and conductivity measurements of incubation medium with the use of pH meter/conductometer (CPC-511 Elmetron, Zabrze, Poland) were performed.

#### 2.2.1. Scanning Electron Microscopy

A Nova NanoSEM 200 scanning electron microscope (SEM; FEI, Eindhoven, The Netherlands) coupled with a Genesis XM X-ray microanalysis system (EDAX, Tilburg, The Netherlands) featuring the Sapphire Si(Li) energy dispersive X-ray (EDX) detector was used to perform the detailed examination of the microstructure of the produced materials. The measurements and observations were conducted in high vacuum conditions, with back scatter electron detector (BSE) at an accelerated voltage of 10–18 kV. The samples were coated with a carbon layer.

#### 2.2.2. Surface Properties

The surface wettability was evaluated by static water contact angle measurements. The contact angle was determined by the sessile drop method with an automatic drop shape analysis (DSA) system, DSA 10 Mk2 (Kruss GmbH, Hamburg, Germany). Ultrahigh quality (UHQ) water droplets of 0.25 μL were applied on each pure and dry sample. The experiments were carried out in constant temperature and humidity conditions.

The arithmetical mean roughness (Ra) was evaluated using a contact profilometer HOMMEL-ETAMIC T1000 wave (Jenoptik AG, Jena, Germany).

The static water contact angle and arithmetical mean roughness values were calculated as an average of 10 measurements and were expressed as the mean ± standard deviation (SD).

#### 2.2.3. Tensile Test

The tensile strength (σM) and Young’s modulus (Et) were determined using a universal testing machine, the Inspect Table Blue 5 kN with a 5-kN load cell (Hegewald&Peschke, Nossen, Germany). The pre-load force was 1 N and the test speed was 50 mm min−1. The samples for measurements were prepared according to EN ISO 527-1 [26]. The mechanical parameters were calculated by averaging 10 measurements and expressed as the mean ± SD.

#### 2.2.4. In Vitro Studies—Bactericidal Efficacy Tests

The tests were performed using two strains of *Staphylococcus aureus* G(+) and two strains of *Escherichia coli* G(−) obtained from the Laboratory of Microbiology of the Provincial Hospital in Gdansk, Poland, and two reference strains, ATCC 13420 and ATCC 25922. *Staphylococcus aureus* and *Escherichia coli* strains had previously been tested for resistance to antibiotics (Table 1). The bacteria strains and their resistance to antibiotics were determined by the disc-diffusion susceptibility test. The disc-diffusion test was performed according to the guidelines of the National Committee for Clinical Laboratory Standards (NCCLS). The disc-diffusion tests were purchased from OXOID Co, Basingstoke, UK. All experiments were performed in triplicate. One type of polymer discs containing silver nanoparticles AgNPs 45T (0.1%) and without silver nanoparticles (as a control) were tested to establish the bactericidal efficacy, using the modified method according to the ASTM E 2180-07 norm (*Standard Test Method for Determining the Activity of Incorporated Antimicrobial Agent(s) In Polymeric or Hydrophobic Materials*) [27]. The only alteration was fitting the norm to the size of the polymer samples. Every sample was tested according to the method described by Ziąbka et al. [21].

#### 2.2.5. In Vitro Tests—Cell Response

In vitro biological evaluation of the materials was carried out using human osteosarcoma cell line Saos-2 (ATCC, Manassas, VA, USA) as well as regular human osteoblasts (NHOst; Lonza, Walkersville, MD, USA). Saos-2 and NHOst cells were expanded in 75 cm^2^ tissue culture flasks (Nunc, Roskilde, Denmark) in McCoy’s 5A medium (ATCC, Manassas, VA, USA) containing 15% foetal bovine serum (FBS; HyClone, San Angelo, TX, USA) and complete osteoblast growth medium (OGM) BulletKit (Lonza, Walkersville, MD, USA) containing 10% FBS, 0.1% ascorbic acid, and 0.1% GA-1000 (Gentamicin Sulfate and Amphotericin-B), respectively, at 37 °C in a humidified, 5% CO_2_ atmosphere. The medium was changed every three days until a 70% confluent cell monolayer developed. Then, cells were detached from culture flasks using 5% Trypsin-EDTA (HyClone, San Angelo, TX, USA), centrifuged, and resuspended in fresh media. Next, 1 mL of the resulting cell suspension at a density of 10^4^ cells/mL was added to the wells of 48-well culture plates (Nunc, Roskilde, Denmark) containing sterile discs of the materials. Saos-2 and NHOst cells were cultured in direct contact with materials in McCoy’s 5A medium (ATCC, Manassas, VA, USA) and complete osteoblast growth medium (OGM) supplemented with differentiation kit SingleQuots (Lonza, Walkersville, MD, USA), containing hydrocortisone-21-hemisuccinate and β-glycerophosphate. The bottom surfaces of tissue culture polystyrene (TCPS) wells served as a control.

ToxiLight 100% Lysis Reagent set (Lonza, Walkersville, MD, USA) was used to disintegrate the cytomembrane of the cultured cells. Then, ToxiLight Bioassay Kit (Lonza, Walkersville, MD, USA) was used in order to establish the entire number of cells confirming their proliferation. The amount of the released adenylate kinase was assessed with a PolarStar Omega reader (BMG Labtech, Ortenberg, Germany). Furthermore, the metabolic activity of the cells was evaluated using the PrestoBlue® Cell Viability Reagent (Invitrogen, Waltham, MA, USA). PrestoBlue® reagent was added to each well with the cells spread on TCPS, after which the materials were incubated for 2 h at 37 °C in a 5% CO_2_ moistened atmosphere. Fluorescence was measured at 560/590 nm (excitation/emission, respectively) using a POLARstar Omega microplate reader (BMG Labtech, Ortenberg, Germany). The results are expressed as the mean ± SD from eight measurements for each group.

The cytotoxicity level of the materials was assessed using ToxiLight Bioassay Kit (Lonza, Walkersville, MD, USA), which measures the release of the enzyme adenylate kinase (AK) from the damaged cells. The luminescence was measured with a PolarStar Omega plate reader (BMG Labtech, Ortenberg, Germany). The test was conducted on the supernatant from the cell culture and the results were compared to the entire enzyme concentration (proportional to the entire number of cells) released from all the cells. The results are expressed as the mean ± SD from eight measurements for each group.

The measurement of alkaline phosphatase (ALP) activity is based on the hydrolysis reaction of 4-MUP (4-methylumbelliferyl phosphate, the substrate for ALP expressed by differentiated osteoblasts), to highly fluorescent product 4-MU (4-methylumbelliferone). After 7 and 14 days of culture, NHOst cells were disrupted via a cyclic freezing/thawing in order to release intracellular ALP. Cell lysates in triplicates were transferred to OptiPlate-96 microplate (PerkinElmer, Waltham, MA, USA) and incubated with equal volumes of 4-MUP liquid substrate system (Sigma-Aldrich, Saint Louis, MO, USA) solution for 1 h. The fluorescence value was determined at 360/440 nm (excitation/emission wavelengths) using PolarStar Omega reader (BMG Labtech, Ortenberg, Germany). The results were compared to the entire enzyme concentration (proportional to the entire number of cells) released from all the cells. The results are expressed as the mean ± SD from eight measurements for each group.

A Nova NanoSEM 200 scanning electron microscope (SEM; FEI, Eindhoven, The Netherlands) was used to perform a detailed morphological examination of the cells adhered to the investigated materials. The observations were conducted in high vacuum conditions, with a back scatter electron detector (BSE) at an accelerated voltage of 10–18 kV. After seven days of cell culture, the materials were rinsed with phosphate buffered saline (PBS) and then the cells were fixed with 3% glutaraldehyde solution in sodium cacodylate buffer at pH 7.4 (POCh, Gliwice, Poland) for 0.5 h. Subsequently, the cells were dehydrated in a graded series of ethanol solution (70%, 80%, 90%, 96%, and 100%) and dried in air. The cell morphologies were evaluated after coating with carbon.

#### 2.2.6. Statistical Analysis

The results were analyzed using one-way analysis of variance (ANOVA) with Duncan post hoc tests, which were performed with Statistica 10 (StatSoft^®^, Tulsa, OK, USA) software. The results were considered statistically significant when *p* < 0.05.

## 3. Results and Discussion

**Figure 1:**
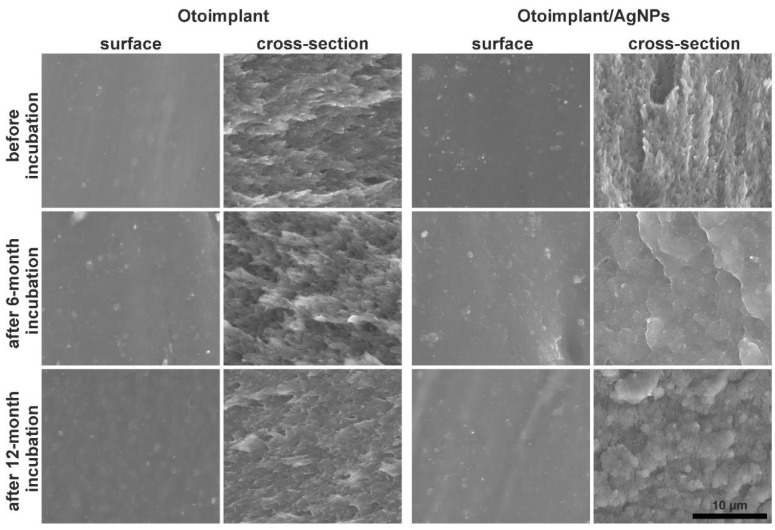
Scanning electron microscope (SEM) images of the surfaces and cross-sections of the Otoimplant and Otoimplant/AgNPs before and after 6- and 12-month incubation in deionized water.

Observations of the surface microstructure of the samples for Otoimplant and Otoimplant enriched with silver nanoparticles showed no significant differences (Figure 1). In the case of nanodioxide-modified material, evenly spaced bright points responsible for the homogeneous distribution of silver nanoparticles in the polymer matrix were noticed both on the surface and in the cross-section. At the same time, the nanoparticles filled the porous structure, which was visible for the non-modified implant. Incubation in distilled water did not cause microstructural changes on the surface and in the cross-section of the pure Otoimplant. However, in the case of a composite prosthesis after 6 and 12 months of incubation, a significant change was seen in the cross-sections. The observed microstructure of cross-sections was similar to a sponge and had clearly rounded shapes, the presence of which resulted from the leaching of silver nanoparticles. Such a phenomenon was consistent with the roughness parameters and our previous results [28].

**Figure 2:**
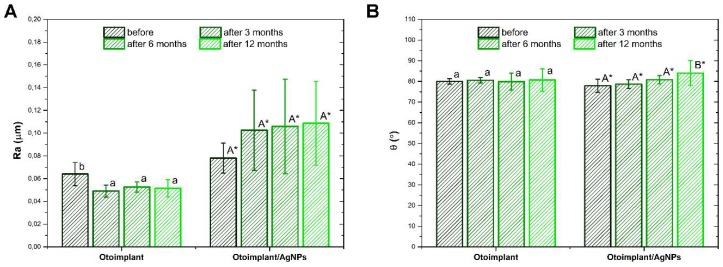
Surface properties of the Otoimplant and Otoimplant/AgNPs before and after 3-, 6-, and 12-month incubation in deionized water: average roughness Ra (**A**) and static water contact angle (**B**). Statistically significant differences (*p* < 0.05) between specific incubation times are marked a–b for Otoimplant and A–B for Otoimplant/AgNPs, respectively. Statistically significant differences (*p* < 0.05) between Otoimplant and Otoimplant/AgNPs at specific incubation time are marked *.

The surface roughness of the materials both before as well as after 3, 6, and 12 months of incubation in deionized water was evaluated on the basis of the Ra parameter (Figure 2A). The surface of the AgNPs-modified material prior to the incubation was characterized by a significantly higher roughness compared with the surface of the polymeric material. Before incubation, the Ra parameter value for the polymer material was less than 0.074 μm, while for the AgNPs modified composite, it did not exceed 0.090 μm. The obtained results indicated low surface roughness of both materials. In the case of Otoimplant, incubation in deionized water led to a statistically significant decrease in the Ra parameter, which remained at a similar level throughout the incubation period. On the other hand, the surface roughness of Otoimplant/AgNPs tended to increase after three months of incubation, and after subsequent periods, the values of the Ra parameter still showed an upward trend, yet they did not exceed 0.147 μm. However, the changes were not statistically significant. The standard deviations of the mean values of the Ra parameter recorded for Otoimplant/AgNPs after incubation in deionized water were significantly higher as compared with the results obtained for the polymer material, which indicates the increased surface inhomogeneity. An increase in the average value of the Ra parameter and standard deviation may indicate the unveiling of AgNPs on the surface of the composite material.

The hydrophilic nature of the surface of the materials both before and after incubation in deionized water was determined by measuring the static contact angle (Figure 2B). Introducing AgNPs to the polymer matrix caused a decrease in surface wettability; nevertheless, the surfaces of both materials showed a hydrophilic character. In the case of Otoimplant, incubation in deionized water did not lead to changes in the contact angle value. On the other hand, the values recorded for Otoimplant/AgNPs during the incubation showed an upward trend, but after 12 months, they did not exceed 90°.

**Figure 3:**
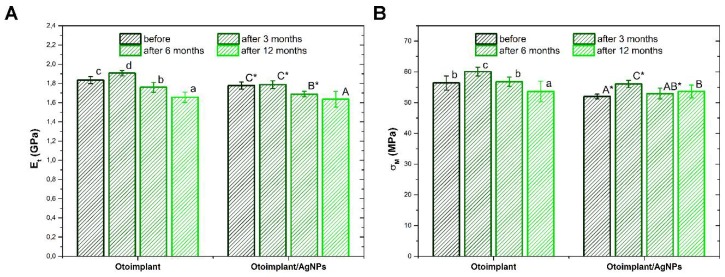
Mechanical properties of the Otoimplant and Otoimplant/AgNPs before and after 3-, 6-, and 12-month incubation in deionized water: Young’s modulus E_t_ (**A**) and tensile strength σ_M_ (**B**). Statistically significant differences (*p* < 0.05) between specific incubation times are marked a–d for Otoimplant and A–C for Otoimplant/AgNPs, respectively. Statistically significant differences (*p* < 0.05) between Otoimplant and Otoimplant/AgNPs at specific incubation time are marked *.

The values of Young’s modulus and tensile strength of materials before as well as after 3, 6, and 12 months of incubation in deionized water are presented in Figure 3A,B. The presence of the monomer in the polymer matrix resulted in a slight reduction of the analyzed mechanical parameters. In the case of Otoimplant, after three months, a statistically significant increase in the value of Et and σM was noted, followed by a gradual decrease. Considering Young’s module for Otoimplant/AgNPs, the gradual decrease in both parameters was observed only from the sixth month of incubation. In turn, the tensile value determined for the composite material increased significantly after three months, and then returned to the level before incubation and did not change significantly until the twelfth month.

**Figure 4:**
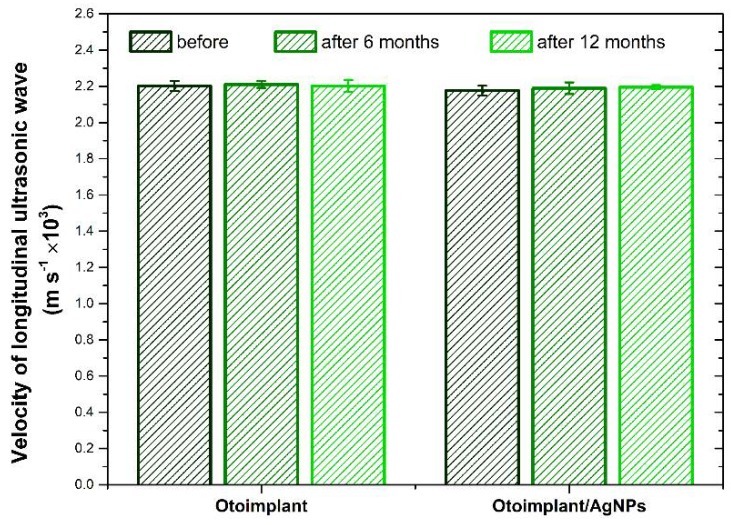
Velocity of longitudinal ultrasonic wave for the Otoimplant and Otoimplant/AgNPs before and after 3-, 6-, and 12-month incubation in deionized water. No statistically significant differences (*p* < 0.05) were detected.

Velocity values of propagation of longitudinal ultrasonic waves in the tested materials before and after incubation in deionized water are shown in Figure 4. The results indicate that both the presence of AgNPs modifier as well as long-term incubation of materials in deionized water did not significantly influence the analyzed parameter.

**Figure 5:**
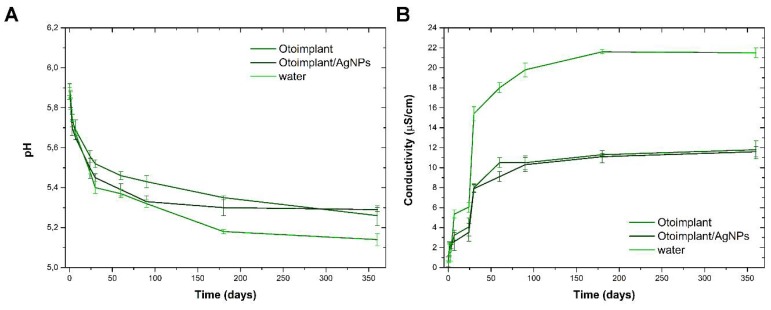
pH (**A**) and conductivity (**B**) changes of deionized water during 3-, 6-, and 12-month incubation of the Otoimplant and Otoimplant/AgNPs.

Changes in the pH and conductivity of deionized water during long-term incubation of the materials are shown in Figure 5A,B. The incubation of both materials resulted in a slight decrease in the pH value of the incubation medium. The same dependence was also noted for the control medium (clean water), the drop was clearly higher. Such results correlated with a slight increase in the conductivity of the medium. The largest changes in both parameters were recorded in the first 30 days of incubation. These changes were related to the dissolution of CO_2_ coming from the atmospheric air in the incubation medium during measurements.

The antibacterial properties of Otoimplant and Otoimplant/AgNPs materials are shown in Table 2. The results indicate that the introduction of AgNPs to the matrix inhibited the growth of all the tested bacterial strains, both the *E. coli* species, representing the Gram-negative bacterial group, as well as the *S. aureus* species belonging to the Gram-positive bacterial group. Growth was inhibited from 86% to 96% for *E. coli* and from 96% to 100% for *S. aureus*. It is noteworthy that the strains of bacteria sensitive to the introduced silver nanoparticles were both sensitive and resistant to antibiotics (Table 2). The addition of 0.4% AgNPs to the Otoimplant material resulted in a bactericidal effect and 100% bacterial mortality (data not shown).

The observed slight differences in the degree of growth inhibition of Gram-negative bacteria as compared with Gram-positive bacteria resulted from the differences in the bacteria cell wall structure. Gram-positive bacteria display stronger activity as peptidoglycan represents 50–90% of cell wall constituents, which means that only one layer protects the cytoplasm. Gram-negative bacteria are characterized by a more complicated structure of the cell wall. Namely, there is a thin layer of peptidoglycan (5–20% cell wall components) between the outer membrane and cytoplasmic membrane, thus the cytoplasm is protected by two layers. Furthermore, the cell wall of *E. coli*, which consists of lipids, proteins, and lipopolysaccharides (LPS), is an effective agent protecting it against biocides, while the cell wall of *S. aureus* does not contain LPS, which means easier elimination of these bacteria [29,30].

**Figure 6:**
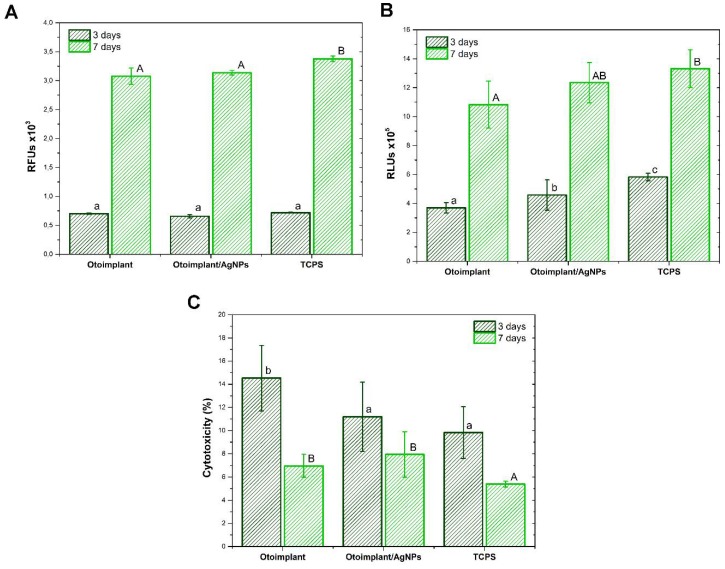
Saos-2 cell response after three and seven days of culture in direct contact with the Otoimplant and Otoimplant/AgNPs: metabolic activity (**A**), the relative cell number (**B**), and cytotoxicity of materials (**C**). Statistically significant differences (*p* < 0.05) between the tested materials are marked a–c for three-day culture and A–B for seven-day culture, respectively. TCPS—tissue culture polystyrene. RFUs—relative fluorescence units. RLUs—relative light units.

The metabolic activity of osteoblastic cells of Saos-2 and their relative abundance after three and seven days of culture in direct contact with materials is presented in Figure 6A,B. The increase in metabolic activity and the number of cells between three and seven days of cultivation indicated a high level of their proliferation in contact with the tested materials, as well as the material at the bottom of the well (TCPS). The metabolic activity of osteoblasts grown for three and seven days on both materials was at a similar level and, at the same time, significantly lower than those deposited on TCPS after seven days of culture. In turn, the number of Saos-2 cells in contact with the AgNPs-modified material was significantly higher in relation to the polymeric material and, at the same time, lower than the values recorded for the TCPS control material. These results correlated with the cytotoxicity of materials (Figure 6C). Cytotoxicity decreased significantly after the prolonged time of cell culture. The highest level of cytotoxicity was noted for Otoimplant after three days, but still it did not exceed 18%. After seven days, the cytotoxicity of both materials was at a similar level and did not exceed 11%, and, at the same time, was significantly higher compared with the values recorded for TCPS.

**Figure 7:**
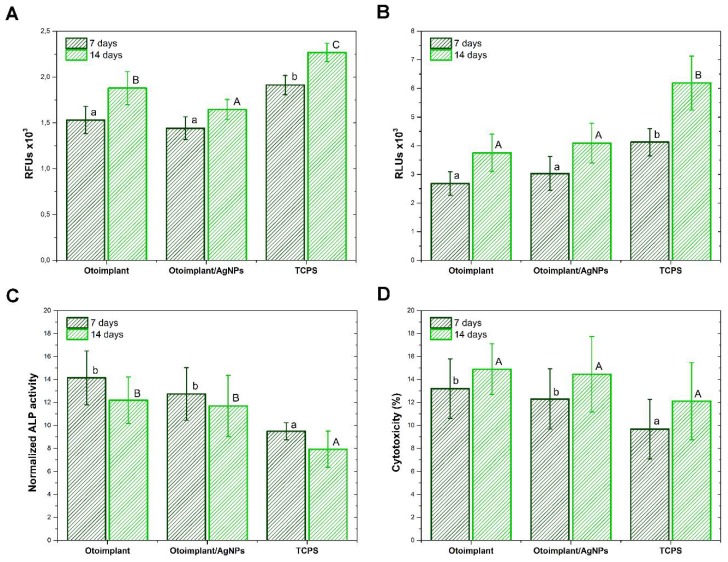
NHOst response after 7 and 14 days of culture in direct contact with the Otoimplant and Otoimplant/AgNPs: metabolic activity (**A**), the relative cell number (**B**), normalized alkaline phosphatase (ALP) activity (**C**), and cytotoxicity of materials (**D**). Statistically significant differences (*p* < 0.05) between the tested materials are marked a–b for 7-day culture and A–C for 14-day culture, respectively.

The metabolic activity of normal human osteoblasts and their relative abundance after 7 and 14 days of culture in direct contact with materials are presented in Figure 7A,B. NHOst cultured on Otoimplant/AgNPs showed reduced levels of metabolic activity against Otoimplant, after 14 days of culture. In turn, the highest activity was recorded for cells deposited on the material at the bottom of the well after both culture periods. The number of osteoblasts in contact with the Otoimplant and Otoimplant/AgNPs after both culture times was significantly lower compared with the values recorded for TCPS. In addition, for all the materials, there was an increase in metabolic activity and cell numbers after the prolonged cell culture. However, it was not as large as in the case of osteoblastic Saos-2 cells, for which the culture was carried out for a shorter period, that is, three and seven days. In particular, the highest increase in NHOst between 7 and 14 days was observed for the control material. The decreased proliferation of osteoblasts cultured in contact with the tested materials was probably caused by the process of their differentiation, which was further indicated by ALP activity—an early marker of osteogenic cell differentiation (Figure 7C). Its level after both cultivation times was significantly higher for Otoimplant and Otoimplant/AgNPs as compared with TCPS. For all the materials, a slight increase in the level of cytotoxicity was observed between days 7 and 14 of cell culture (Figure 7D). After seven-day culture, the Otoimplant and Otoimplant/AgNPs materials exhibited significantly higher cytotoxicity compared with the TCPS control material. Yet the cytotoxicity values did not exceed 18% after 14 days and they were not statistically significant.

**Figure 8:**
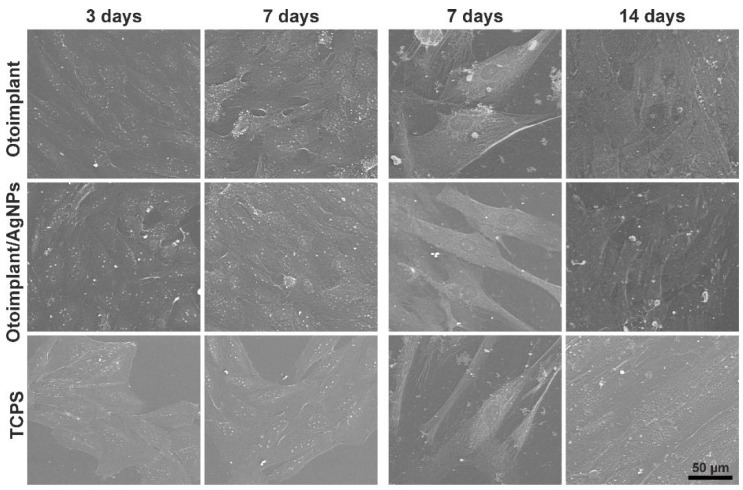
SEM images of Saos-2 cells after three and seven days of culture and NHOst after three and seven days of culture in direct contact with the Otoimplant, Otoimplant/AgNPs, and TCPS. Magnification 1000×.

The morphology of osteoblastic cells of Saos-2 and of regular human osteoblasts in contact with the materials was evaluated on the basis of SEM photomicrographs (Figure 8A,B). Both types of cells deposited on the materials displayed the correct morphology and had numerous cytoplasmic protrusions ensuring cell–cell and cell–material contact. Cells evenly covered the entire surface of the materials, showing a high degree of adhesion/flatness. After longer times of culturing, the cells covered almost the entire surface of the tested materials, which confirms the high level of their proliferation.

The research involving osteoblastic cells of the Saos-2 line derived from human osteosarcoma was aimed at assessing the initial cytocompatibility of the obtained materials. This line is often chosen as a cell model in the in vitro biomaterials testing. The tests run on regular human osteoblasts in a medium with differentiating factors were primarily aimed at the initial assessment of differentiation capability in contact with the materials. This property may improve the implant’s integration with bone tissue, prevent destabilization of the implant, and ensure a more effective sound wave transmission in the form of vibrations.

The differentiated response of osteoblastic cells of Saos-2, as well as regular human osteoblasts in contact with the obtained materials and the material at the bottom of the well (TCPS) may result primarily from diversified topography and surface roughness. The literature unambiguously indicates that surfaces with higher roughness, both on the micro- and nanometric scale, favour the adhesion of precursor cells and osteoblasts in vitro, as well as the implant-bone integration in the in vivo conditions [31,32,33,34,35,36]. A larger development of the material surface, that is, higher roughness, may promote the adsorption of proteins involved in the cells adhesion (e.g., fibronectin) [31]. Surface roughness also affects the process of osteogenic differentiation. Liao et al. demonstrated that osteoblasts cultured in contact with the micro-structured surface of polydimethylsiloxane (PDMS) were characterized by a significantly higher level of ALP activity and extracellular matrix mineralization as compared with the cells in contact with the smooth-surfaced PDMS [32]. The analysis carried out for osteoblasts cultured in contact with the titanium surface subjected to a differentiated treatment showed that the roughness increase (0.6 μm < Ra < 5.2 μm) had a significant effect on the expression of a number of genes related to osteogenesis [37]. You et al. proved that the nanostructured surface of polyurethane in combination with differentiating agents present in the culture medium greatly accelerates the osteogenic differentiation of human mesenchymal stem cells, causing an increase in ALP activity and expression of osteocalcin and osteopontin [38].

## 4. Conclusions

The in vitro biological behaviour of polymeric prostheses enriched with silver nanoparticles for ossicles reconstruction was investigated. The tests evaluated its bactericidal efficacy, cytotoxicity, metabolic activity, and biostability. It has been proven that the silver nanoparticles distribution in the polymeric matrix plays an important role with regard to bacteria and cells adhesion. It also affects morphology, topography and wettability of materials. Incorporation of nanoparticles increases material roughness and improves hydrophilicity. It has been stated that even a small amount of nanoadditive may cause a bactericidal effect. The results indicate that 0.1% wt. of silver nanoparticles is enough to eradicate bacteria and guarantee biocompatibility. Such a low content of nanoparticles is enough to ensure a constant level of advantageous mechanical parameters. We have revealed the stability of the tensile strength and Young’s modulus, which is crucial for longevity of a medical device. Finally, in vitro biostability of our prosthesis has been proven via the measurements of pH, conductivity, and propagation of longitudinal ultrasonic waves.

## Figures and Tables

**Table 1:** Antibiogram pattern of *Staphylococcus aureus* and *Escherichia coli* strains used in the study, isolated from patients.

Bacteria	Antibiotics (Diameter of Zone, mm)																
	CIP	OX	GN	Va	SXT	E	L	P	TEC	AMC	AMP	KZ	CXM	CTX	CAZ	PIP	AK
**Sensitive strain of *Staphylococcus aureus***																	
1521—from blood	25 s	19 s	20 s	16 s	29 s	26 s	26 s	13 s	15 s	21 s	nt	nt	nt	nt	nt	nt	nt
**Resistant strain of *Staphylococcus aureus***																	
703/k—from wound infection	21 s	6 r	20 s	17 s	27 s	6 r	6 r	6 r	16 s	6 r	nt	nt	nt	nt	nt	nt	nt
**Sensitive Strain of *Escherichia coli***																	
1285—from urine	27 s	nt	19 s	nt	25 s	nt	nt	nt	nt	20 s	19 s	18 s	18 s	28 s	26 s	23 s	20 s
**Resistant strain of *Escherichia coli***																	
4162—from cerebrospinal fluid	28 s	nt	6 r	nt	6 r	nt	nt	nt	nt	20 s	6 r	15 ss	17 ss	22 ss	17 ss	14 r	6 r

r—resistant, s—sensitive, ss—semi-sensitive, nt—not-tested, CIP—ciprofloxacin, OX—oxacillin, GN—gentamicin, Va—vancomycin, SXT—sulfamethoxazole/trimethoprim, E—erythromycin, L—lincomycin, P—penicillin, TEC—teicoplanin, AMC—amoxicillin with clavulanic acid, AMP—ampicillin, KZ—cephazolin, CXM—cefuroxime sodium, CTX—cefotaxime, CAZ—ceftazidime, PIP—piperacillin, AK—amikacin.

**Table 2:** Antimicrobial efficacy of the Otoimplant and Otoimplant/AgNPs. CFU – colony-forming unit.

Bacteria Strain	Otoimplant	Otoimplant/AgNPs	% of Growth Inhibition of Bacteria Growing on Otoimplant/AgNPs
***Escherichia coli* (Gram-negative) [CFU]**			
Reference strain ATCC 25922	5.5 × 10^3^ ± 6 × 10^2^	6 × 10^2^ ± 1 × 10^2^	89.09 ± 0.56
Sensitive strain no 1285	2.3 × 10^4^ ± 3.6 × 10^3^	3.2 × 10^3^ ± 2.5 × 10^2^	86.08 ± 0.95
Resistant strain no 4162	3.8 × 10^4^ ± 1.5 × 10^3^	1.5 × 10^3^ ± 5 × 10^2^	96.05 ± 1.12
***Staphylococcus aureus* (Gram-positive) [CFU]**			
Reference strain ATCC 13420	3.6 × 10^3^ ± 7 × 10^2^	5 ± 1	99.86 ± 0.01
Sensitive strain no 1521	4.1 × 10^4^ ± 1.3 × 10^3^	0 ± 0	100 ± 0.00
Resistant strain no 703/k	2.7 × 10^4^ ± 2.5 × 10^3^	9.5 × 10^2^ ± 5 × 10^1^	96.48 ± 0.13
